# Investigation of intake air temperature effect on co-combustion characteristics of NH_3_/gasoline in naturally aspirated high compression ratio engine with sub-chamber

**DOI:** 10.1038/s41598-023-38883-3

**Published:** 2023-07-19

**Authors:** Emir Yilmaz, Mitsuhisa Ichiyanagi, Qinyue Zheng, Bin Guo, Narumi Aratake, Masashi Kodaka, Hikaru Shiraishi, Takanobu Okada, Takashi Suzuki

**Affiliations:** 1 Department of Engineering and Applied Sciences Faculty of Science and Technology, Sophia University, Tokyo, 102-8554 Japan; 2 Graduate School of Science and Technology, Sophia University, Tokyo, 102-8554 Japan

**Keywords:** Engineering, Mechanical engineering

## Abstract

Recently, ammonia (NH_3_), which has a higher energy density than hydrogen, has gained attention for zero-carbon emission goals in the transportation sector. However, in a conventional internal combustion engine (ICE), NH_3_ combustion mechanism is still under investigation. In this paper, to further expand the knowledge on the adoption of NH_3_ in ICEs, authors conducted NH_3_/gasoline co-combustion experiments in a modified, 17.7:1 compression ratio, naturally aspirated spark-assisted CI engine with sub-chamber. The sub-chamber was chosen in order to enhance the combustion speed of NH_3_. In addition, the sub-chamber was equipped with glow and spark plugs to overcome the high auto-ignition temperature of NH_3_. Engine performance and NO_X_ emissions were studied under three different intake air temperatures. During the experiments, NH_3_ content was increased gradually where the engine was run under lean conditions. Although higher NH_3_ content was achieved compared to our previous work, increasing the intake air temperature resulted in decreased charging efficiency. In addition, corrosion was found on the piston ring after 120 h of operation, negatively affecting the engine performance. Furthermore, NH_3_/gasoline co-combustion duration was shortened drastically with the influence of the sub-chamber, where the longest combustion duration under the present conditions was found to be 17°CA.

## Introduction

In regard with the recent news on the European Union changing its original plan of phasing out internal combustion engines (ICEs), unconventional fuels (such as ammonia (NH_3_), hydrogen (H_2_), synthetic fuels (E-fuels) etc.) are gaining popularity in the ICEs research. It will now be possible to sell new ICE vehicles in Europe using carbon–neutral fuels^1^. From these, NH_3_ is a strong candidate for further expanding its usage in various industries. It can be used in energy storage due to its high hydrogen content, and also in the transportation sector as a fuel for power generation^2,3^. As can be seen from its structure, NH_3_ does not include any carbon atoms, in which there are not any CO_2_ emissions, thus regarded as a carbon-free fuel. Some important points that need to be addressed are the toxicity and NO_X_ emissions at elevated temperatures due to the nitrogen atom (N) available. However, conventional selective catalytic reduction (SCR) system is capable of lowering NO_X_ emissions substantially if the SCR-inlet temperature is maintained at 200°C^4^.

Table 1 shows selected various properties of NH_3_ and its comparison to hydrogen and gasoline. When compared with hydrogen, NH_3_ has higher volumetric energy density, however, when compared with gasoline, it still has around 30% less volumetric energy density. In addition, numerous studies have been going on about utilization of H_2_ and NH_3_ in ICEs. Kim et al. used direct injection method of H_2_ with different mixture formation modes^5^. Furthermore, since NH_3_ has lower flame propagation speed, a more common approach was to utilize it in marine engines with lower engine speeds^6^. In order to use NH_3_ in passenger vehicles, where higher engine speed is needed, its properties need to be considered. As shown in Table 1, NH_3_ has high octane rating and high latent heat of vaporization, which allows its usage in an engine with high compression ratio (CR). In-line with this knowledge, Pochet et al. conducted ammonia-hydrogen dual-fuel combustion studies in an engine with CR of 15:1^7^, 16:1^8^, 22:1^9^ under Homogenous Charge Compression Ignition (HCCI) mode. Lhuillier et al. conducted experiments using NH_3_ and hydrogen blends in a spark ignition (SI) engine with CR of 10.5^10^. They also showed that phasing of the combustion is correlated with the Laminar Burning Velocity (LBV) of the mixture under SI timing conditions. In a more recent study, Mounaim-Rousselle et al. performed experiments on a spark-assisted compression ignition (CI) single cylinder ICE with CR between 14 and 17, running on pure NH_3_^11^. They managed to obtain stable combustion at low loads and various engine speeds, proving that high CR and spark-ignition method works well for NH_3_ as fuel.

**Table 1:** NH_3_, Hydrogen and Gasoline properties and their comparison at 300 K and 0.1 MPa.

	Ammonia^2,5,15,19^	Hydrogen^16,18^	Gasoline^5,16,20^
LBV (m/s) [@ $\phi =1$]	0.07	3.51	0.58
Octane rating, RON	> 130	> 100	90–98
Minimum auto ignition temperature (K)	930	773	503
Adiabatic flame temperature (K)	2090	2390	2411
Minimum ignition energy (mJ)	8	0.018	0.14
Flammability limit in air (vol.%)	15–28	4.7–75	0.6–8
Latent heat of vaporization (kJ/kg)	1167	461	300
Stoichiometric A/F mass ratio	6.1	34	14.5
Volumetric energy density (GJ/m^3^)	11.3	4.7	33

In-line with these previous studies, the authors believed that a novel engine design is necessary to adopt NH_3_ as an ICE fuel due to its disadvantages. It has been proven that a sub-chamber can shorten the duration of combustion in SI engines^12^. Thus, a sub-chamber equipped with a spark plug and a glow plug was added to the experimental engine, where an air heater mechanism was also added to the intake system of the experimental engine. By increasing the temperatures inside engine cylinder, the aim was to promote NH_3_ combustion and increase its content in the fuel mixture. The overall purpose of these consecutive studies is to develop an ICE, which can run on alternative fuels to reduce dependency on gasoline and further decrease CO_2_ emissions. The present study used a combination of two methods in-line with previous studies^11,13^, where the engine experiments were conducted in a high compression ratio spark-assisted HCCI engine. However, in our previous study the highest NH_3_ content achieved was only 33%. As compression-ignition process needs high in-cylinder temperatures, the present study is conducted under higher intake air temperatures, up to 348 K. The objective of this study was to investigate the intake temperature effect and further increase the NH_3_ content while attaining stable NH_3_/gasoline co-combustion and gradually decrease the gasoline content in the fuel-mixture. Modified experimental engine performance along with NO_X_ emissions were reported.

## Experimental method and combustion mechanism

### Experimental setup

**Figure 1:**
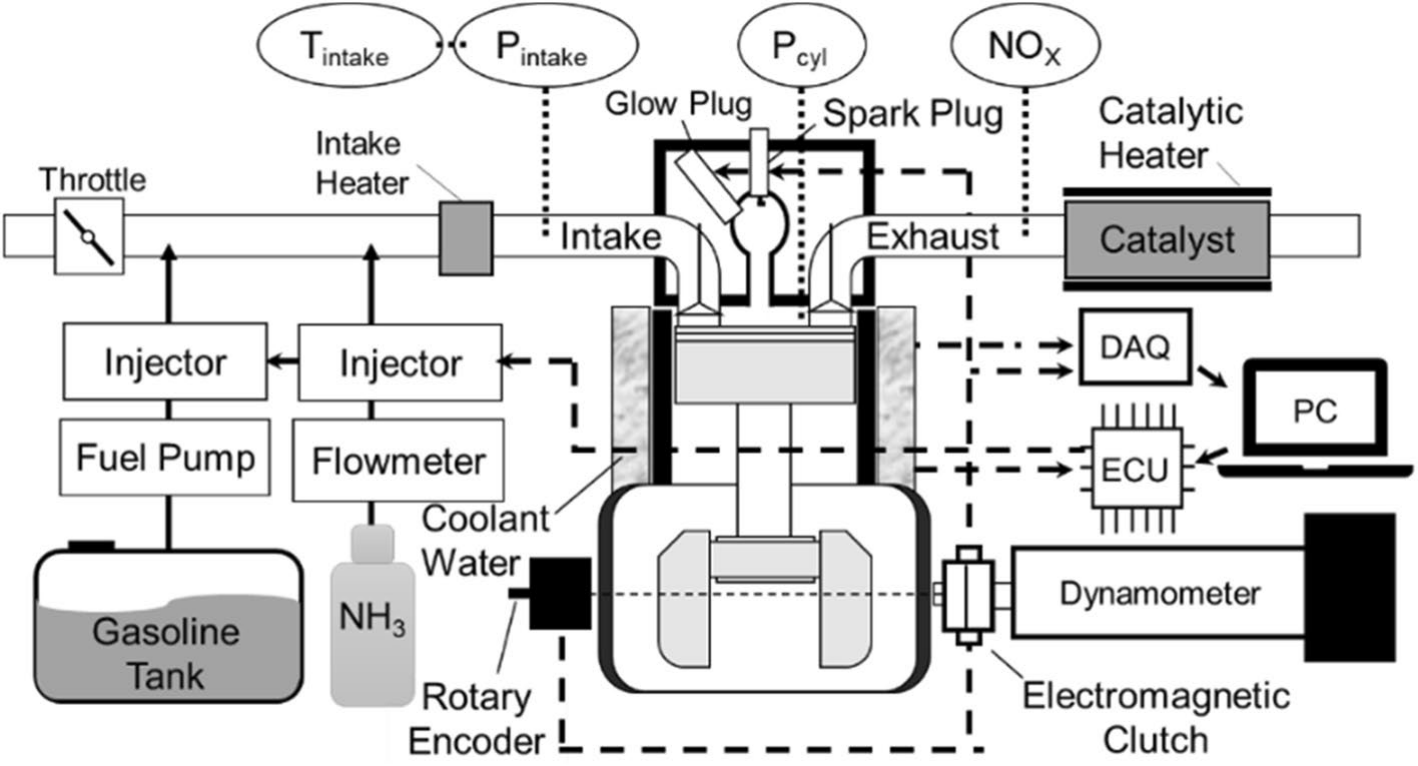
Schematic view of the experimental engine and data acquisition system.

In this study, a high compression ratio (CR:17.7), four stroke, water-cooled horizontal single-cylinder diesel engine (YANMAR TF120V-E2) was modified to conduct co-combustion experiments using NH_3_/gasoline mixture in a spark-assisted HCCI engine. Figure 1 illustrates the schematic view of the experimental setup. The original diesel fuel injector was removed, and cylinder head was machined purposely to create a sub-chamber (23.5 × 10^–6^ m^3^) equipped with spark and glow plugs (NGK-SRM). A gasoline injector (BOSCH INJ-035) and an ammonia injector (Nikki O-RING Type) were installed in the intake port. A pressure sensor was installed in the main chamber with a displacement of 638 × 10^–6^ m^3^. A flat head piston was used to increase the compression ratio with a bore diameter and stroke of 92 mm, and 96 mm, respectively. The connection between the main and the sub-chamber was made through an orifice with a cross-sectional area of 52.6 mm^2^. The throttle valve was controlled by hand to obtain a constant intake pressure, which was measured by the intake port pressure sensor. Fuels (gasoline and NH_3_) were injected into the intake port. Injection timing and fuel quantity were controlled by a general-purpose Engine Control Unit (ECU) (INFINITY SERIES 7). Fuel injection signal and spark ignition signal were transmitted to the data logger, concurrently. Spark ignition was used inside the sub-chamber, where the ignition timings were controlled by the same ECU and adjusted between 350°CA ~ 370°CA depending on the fuel mixture conditions. In addition, piston’s Top Dead Center (TDC) timing was determined by using in-cylinder pressure data, which enabled the authors to confirm the real-time position of the piston. It was used for fuel injection and spark ignition timings. This was done by matching the in-cylinder pressure data to the signal from the rotary encoder (E6B2-CWZ6C) at every two revolutions of the crankshaft. The coolant system of the modified experimental engine was composed of a coolant heater, heat exchanger, and a pump to control the water temperature inside engine’s cooling channels. Table 2 shows the overall engine specifications.

**Table 2:** Modified experimental engine specifications.

Engine model	YANMAR TF120V-E2
Engine type	Water-cooled, four stroke, horizontal single cylinder
Sub-chamber	Spherical swirl with glow plug
Valve mechanism	Overhead valve
Intake valve closed [°CA]	216
Exhaust valve open [°CA]	502
Injection method	Port injection
Ignition method	Spark ignition
Gasoline supply	Gasoline injector
Ammonia supply	Ammonia injector
Total displacement [m^3^]	638 × 10^–6^
Sub-chamber displacement [m^3^]	23.5 × 10^–6^
Bore × Stroke [mm]	92 × 96
Number of orifices [–]	1
Orifice cross-sectional area [mm^2^]	52.6
Compression ratio [–]	17.7

### Experimental conditions

Table 3 shows the experimental conditions used in this study. The modified experimental engine was connected to an eddy current dynamometer through an electromagnetic clutch to control and set the engine speed to 1,000 rpm. Coolant water temperature data was sent to the ECU and a PID controller stabilized the coolant temperature at 343 K. The modified experimental engine was originally a naturally aspirated, where the intake pressure was 99 kPa. The glow plug’s voltage was set to 10 V. A catalyst was installed at the exhaust section of the modified experimental engine as exhaust after treatment and heated to 573 K with catalyst heaters to enable optimum efficiency for the exhaust gas treatment. As shown in Table 3, engine experiments took place by changing the intake air temperature between 298 K ~ 348 K and NH_3_ content in the fuel mixture. The fuel consumption was calculated from the gasoline and NH_3_ injectors’ flow meters, where the total calorific value of fuel was calculated accordingly. The injection ratio was changed depending on how much the calorific value of NH_3_ accounted for the total calorific value. The excess air ratios were altered between 1.17 and 1.22, depending on the NH_3_ content. This was because total calorific values of different ratios of NH_3_/gasoline mixtures were set to same. Under these conditions the highest indicated thermal efficiencies and the lowest coefficient of variation of the indicated mean engine pressure (COV_IMEP_) were attained for the modified experimental engine. Low heating value is used for determining the NH_3_ content. For example, NH_3_ 33% means the remaining 67% is gasoline fuel was used for the experiments. In this experimental setup and conditions, stable NH_3_/gasoline co-combustion was achieved up to 66% of NH_3_ and 34% of gasoline as the injected fuel ratio. Above this limit of NH_3_ content, COV_IMEP_ values were greater than 5%, thus omitted. Only averaged values over 100 cycles were presented in this study, where the investigated operating conditions were summarized in Table 3.

**Table 3:** Experimental conditions.

Engine speed [rpm]	1000
Fuel for injector 1 [–]	Gasoline
Fuel for injector 2 [–]	Ammonia
Gasoline injection timing [°CA]	− 30
Ammonia injection timing [°CA]	10
Ignition timing [°CA]	350 ~ 370
Coolant temperature [K]	343
Intake pressure [kPa]	99
Intake air temperature [K]	298, 323, 348
Glow plug voltage [V]	10
Ammonia content [%] (based on LHV)	31, 38, 45, 52, 59, 63, 66

### Combustion mechanism

As mentioned in the introduction section, this study was done in a high CR engine which was modified for NH_3_/gasoline co-combustion. The combustion mechanism was as follows: Fuels were injected to the intake port during the intake stroke of the engine (gasoline @-30°CA and ammonia @10°CA, as written in Table 3). These fuels were mixed during the intake and compression strokes. During the compression stroke some of the air-NH_3_/gasoline fuel mixture was directed into the sub-chamber, where glow plug was used to heat up the air–fuel mixture, which was mainly used to promote combustion increasing the ambient temperature. The phenomenon was experimentally proven in our previous study^16^. The spark plug was initiated at different CA timings depending on the NH_3_ content inside the for Minimum spark advance for Best Torque (MBT) and its results are discussed in the next section. As the power stroke began (after 360°CA for this study), spark-ignited air–fuel mixture inside the sub-chamber was flown into the main-chamber from the orifice, where, at this point, the remaining air–fuel mixture inside the main-chamber was considered to be well-mixed. This phenomenon started HCCI mode as high temperature combusted gas was flown from the sub-chamber into the main chamber.

### Evaluation method

In-cylinder pressure data was used to calculate the indicated mean effective pressure (IMEP), *P*_*mi*_ [MPa] which was used to characterize the engine's power, *P*_*i*_ [kW]. Equation (1) was used to calculate IMEP which was changed into a discretized form to be used in the data analyses, as Eq. (2).

1 $${P}_{mi}=\frac{1}{{V}_{s}}\cdot \oint PdV$$

2 $${P}_{mi}=\frac{1}{{V}_{s}}\cdot {\sum }_{j=0}^{a-1}\frac{{P}_{j+1}+{P}_{j}}{2}\cdot ({V}_{j+1}-{V}_{j})$$

where *V*_*s*_ is the stroke volume [m^3^], *P* is the in-cylinder pressure [MPa], *V* is the volume of both main and sub-chamber [m^3^]. *P*_*j*_ is the in-cylinder pressure [MPa] and *V*_*j*_ is the volume at per CA, *a* is the total number of data in one cycle [–].

Equation (3) was used to calculate the COV_IMEP_ to confirm the combustion stability. The combustion is considered to be stable when the COV_IMEP_ is less than 10%^11^.

3 $${CO\mathcal{V}}_{IMEP}=\frac{{\sigma }_{{P}_{mi}}}{\overline{{P }_{mi}}}\times 100\%$$

where *σ*_*Pmi*_ is the standard deviation of IMEP [MPa] and $\overline{{P }_{mi}}$ is the average IMEP [MPa].

Generally, during the combustion period, heat is transferred by both convection and radiation between the in-cylinder combustion gas and the cylinder walls. However, the radiative heat transfer in a SI engine only accounts for 3–4% of the total heat transfer^21^, thus omitted in this study. The heat transfer between the combustion gas and cylinder wall through Eq. (4), which is based on Newton’ s law of cooling.

4 $${Q}_{ht}(\theta )={h}_{c}(\theta ){A}_{c}({T}_{(\theta )}-{T}_{w})$$

where *Q*_*ht*_ represents the heat transfer from the combustion gas to the cylinder wall per crank angle [kW/°CA], *A*_*c*_ represents the combustion chamber area [m^2^], *T*_*w*_ is the mean temperature of cylinder wall, which was used as 450 K^22^. *h*_*c*_ is the convective heat transfer coefficient of gas [kW/m^2^∙K], which was expressed in Eq. (5) based on the Hohenberg’s correlation^23^.

5 $${h}_{c}\left(\theta \right)=1879{P(\theta )}^{0.8}{T(\theta )}^{-0.4}{V(\theta )}^{-0.06}{(\overline{{S }_{p}}+c)}^{0.8}$$

where *c* is calibration factor which is equal to 1.4 as suggested by Hohenberg^23^; $\overline{{S }_{p}}$ represents the mean piston speed [m/s] and expressed in Eq. (6).

6 $$\overline{{S }_{p}}=2\cdot S\cdot \frac{n}{60}$$

The cooling loss, *L*_*c*_ [%], was calculated by the Eq. (7).

7 $${L}_{c}=\frac{{\int }_{{\theta }_{IVC}}^{{\theta }_{EVO}}{Q}_{ht}d\theta }{{m}_{a}\cdot {H}_{ua}+{m}_{g}\cdot {H}_{ug}}$$

where lower and upper limit of the integral *θ*_*IVC*_, *θ*_*EVO*_ are the crank angle of the intake valve closed (IVC) and the exhaust valve open (EVO) timings [˚CA], respectively. Timings are given in Table 2.

Equation (8) was used to calculate the heat release rate (HRR) to show the heat release per crank angle.

8 $${Q}_{HRR(i)}=\frac{1}{\kappa -1}\left(V\frac{dP}{d\theta }+\kappa P\frac{dV}{d\theta }\right)+{Q}_{ht}$$

where *Q*_*HRR*_ is the heat energy [J/°CA], subscript (*i*) was used for different intake air temperature cases, *P* is the in-cylinder pressure [MPa], *V* is the volume of both main and sub-chamber [m^3^], *κ* is the specific heat ratio, *θ* is the crank angle in degrees [°CA]. The combustion efficiency [%], *η*_*c*_, was used to determine the burned fuel ratio, which was calculated from the following equation:

9 $${\eta }_{c}=\frac{{\int }_{{\theta }_{1}}^{{\theta }_{2}}{Q}_{HRR(i)}d\theta }{{m}_{a}\cdot {H}_{ua}+{m}_{g}\cdot {H}_{ug}}$$

where *θ*_*1*_ is the crank angle when the fuel chemical energy has started to be released [˚CA] and *θ*_*2*_ is the crank angle when the fuel chemical energy has totally been released [°CA]. *m*_*a*_ and *m*_*g*_ are the mass of injection NH_3_ and gasoline [kg], respectively. *H*_*ua*_ and *H*_*ug*_ are the lower calorific values for NH_3_ and gasoline [kJ/kg], respectively. The lower calorific values used for NH_3,_ and gasoline are 18,600 kJ/kg, and 42,280 kJ/kg, respectively. Normalized mass fraction burned (NMFB) was obtained from Eq. (10) by dividing the instantaneous heat release energies between IVC and EVO timings from three different intake air temperatures to the heat release energy obtained from 298 K case.

10 $$NMFB=\frac{{\int }_{{\theta }_{IVC}}^{{\theta }_{EVO}}{Q}_{HRR(i)}d\theta }{{\int }_{{\theta }_{IVC}}^{{\theta }_{EVO}}{Q}_{HRR(@298K)}d\theta }$$

where integral of *Q*_*HRR(i)*_ with respect to crank angle is the instantaneous heat release energy [J] for each intake air temperature case under NH_3_ content of 59%. Integral of *Q*_*HRR*(298 K)_(*θ*) with respect to crank angle is the heat release energy [J] under the intake air temperature of 298 K. NH_3_ content of 59% was the highest case for all intake air temperatures where stable combustion was achieved, thus used for comparison.

Indicated power, *P*_*i*_ [kW] of the modified engine was calculated with Eq. (11), where these values were used to calculate indicated thermal efficiency *η*_*i*_, as shown in Eq. (12).

11 $${P}_{i}=\frac{{P}_{mi}\cdot A\cdot S\cdot n\cdot Z\cdot i}{60}$$

where *A* is the cylinder cross-sectional area [m^2^], *S* is the piston stroke [m], *n* is the engine speed [rpm], *Z* is the number of cylinders, which was 1 for this study since the experimental engine was a single cylinder type, and *i* is a constant number, which was used as 0.5, since the experimental engine was a four-stroke type.

12 $${\eta }_{i}=\frac{{P}_{i}}{{F}_{a}\cdot {H}_{ua}+{F}_{g}\cdot {H}_{ug}}$$

where *F*_*a*_ and *F*_*g*_ are the fuel consumption rates for the NH_3_ and gasoline [kg/s], respectively.

The in-cylinder gas temperature [K] was calculated by the Eq. (13), which was derived from the ideal gas law.

13 $${T}_{(\theta )}=\frac{{P}_{(\theta )}\cdot {V}_{(\theta )}}{{P}_{in}\cdot {V}_{in}}{T}_{in}$$

where *P*_*(θ)*_, *V*_*(θ)*_ and *T*_*(θ)*_ denote the pressure [MPa], volume [m^3^], and temperature [K] per crank angle of the in-cylinder gas from the IVC to EVO, respectively. *P*_*in*_, *V*_*in*_ and *T*_*in*_ are pressure, volume, and temperature at IVC; thus, *T*_*in*_ is the same as the intake air temperature.

## Results and discussion

### Effect of MBT on NH_3_/gasoline co-combustion

**Figure 2:**
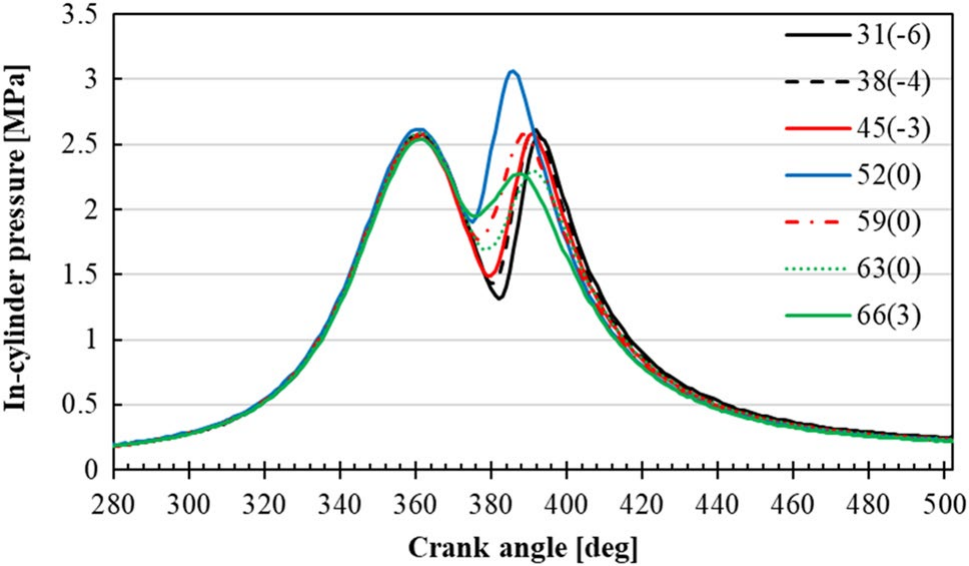
In-cylinder pressure at each MBT of different NH_3_ content under the intake temperature of 348 K (the number in the parenthesis is the MBT at each condition).

During the engine experiments in-cylinder pressure data was measured, where the case of 348 K intake air temperature under different NH_3_ content is shown in Fig. 2. Due to the presence of sub-chamber, in-cylinder pressure graph resulted in two peak points (camelback figure), one from around 360°CA at TDC due to piston’s compression, whereas the second one occurred due to the combustion took place inside the main-chamber during the expansion (power) stroke. As the NH_3_ content increased from 31 to 66%, in-cylinder pressure initially increased, up to 52%, and then gradually decreased with the increasing NH_3_ content. However, peak in-cylinder pressure occurred around the same CA with different NH_3_ content in the fuel mixture due to the different MBT timings. The obtained in-cylinder pressure data for different NH_3_ contents was used to calculate IMEP and COV_IMEP_ under each intake air temperature condition. The highest attained IMEP values were used to calculate the MBT, which was shown in parentheses next to the NH_3_ content in the figures below. As an example, in the case of 31% NH_3_ content at 348 K of intake air temperature, MBT was found to be − 6 deg BTDC (Before-Top Dead Center), meaning MBT was achieved at 6°CA after the piston reached TDC, which corresponds to the beginning of the power stroke. Thus, written as 31(-6). It should be noted that, for the same NH_3_ content under different intake air temperatures, MBT was found to be different.

**Figure 3:**
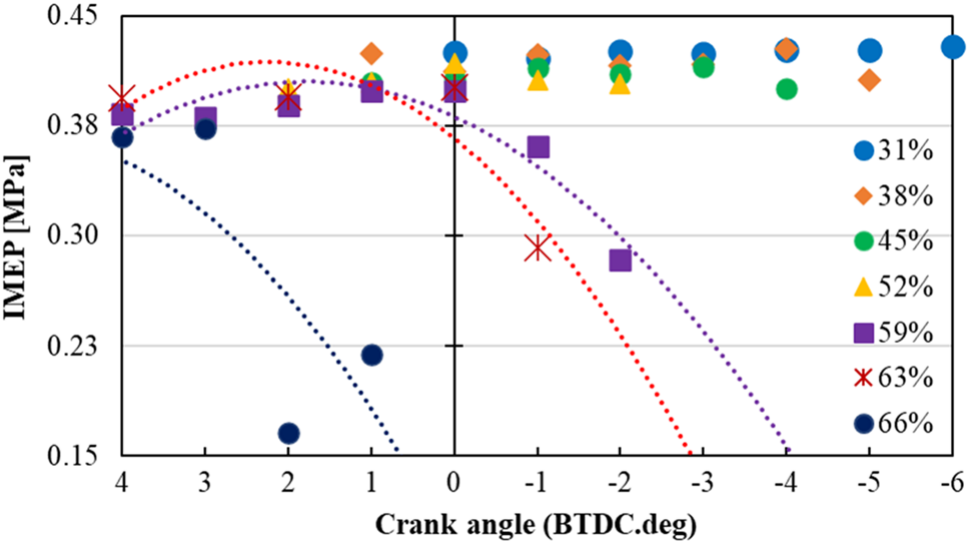
IMEP under the condition of each NH_3_ content with the ignition timings from 4 to − 6 BTDC (λ = 1.17⁓1.22, glow plug voltage: 10 V, intake temperature: 348 K).

Figure 3 illustrates IMEP under 348 K intake air temperature with different NH_3_ content in the fuel mixture. IMEP values were calculated from Eq. (2). As can be seen from this figure, at 31% NH_3_ content, combustion was stable over a long range due to high gasoline content in the fuel mixture. The highest IMEP (MBT) was found to be − 6 deg BTDC for 31% NH_3_ at the intake air temperature of 348 K. However, as the NH_3_ content got higher, the range of stable combustion got narrower. This phenomenon was related to the slow laminar burning velocity of NH_3_^16,17^. It is a known fact that laminar burning velocity of a fuel affects both advanced limit and retard limit of the combustion process. As can be seen from Fig. 3, for the 31% NH_3_ content case, IMEP values were stable for a wider range. However, as the NH_3_ content was increased, both MBT timings, and stable IMEP results were getting narrower. For high NH_3_ content cases, when the ignition time was advanced too much, in-cylinder pressure (due to piston’s position) and in-cylinder gas temperature were lowered. However, it is also known that NH_3_ needs high in-cylinder gas temperature to be ignited. Thus, the flame core of the fuel mixture could not expand properly. On the contrary, once again for high NH_3_ content cases, when the retard limit was extended, this time piston started to move down, lowering both in-cylinder pressure and temperature. At these timings partial burn of the fuel mixture occurred, resulting in lowered IMEP values. Three trendlines were added in Fig. 3, for NH_3_ contents of 59%, 63% and 66%. Reverse parabolic curves depict the effect of advanced and retard limits caused by narrowed stable combustion range for high NH_3_ content cases. At 348 K, the highest NH_3_ content achieved was 66%, where combustion got unstable for ignition timings later than − 1 deg BTDC. It should be noted that, even though the same modified experimental engine was used from our previous study^14^, compared to our previous work the coolant temperature was increased from 318 to 343 K. In addition, engine experiments started with gasoline fuel in lean state (excess air conditions), and then NH_3_ was added gradually into the fuel-mixture. This method was used to overcome the knocking phenomenon and further increase the NH_3_ content in the fuel mixture. These modifications were thought to be the reason behind how higher NH_3_ content was successfully combusted, while attaining COV_IMEP_ values under 5%, for all cases.

**Figure 4:**
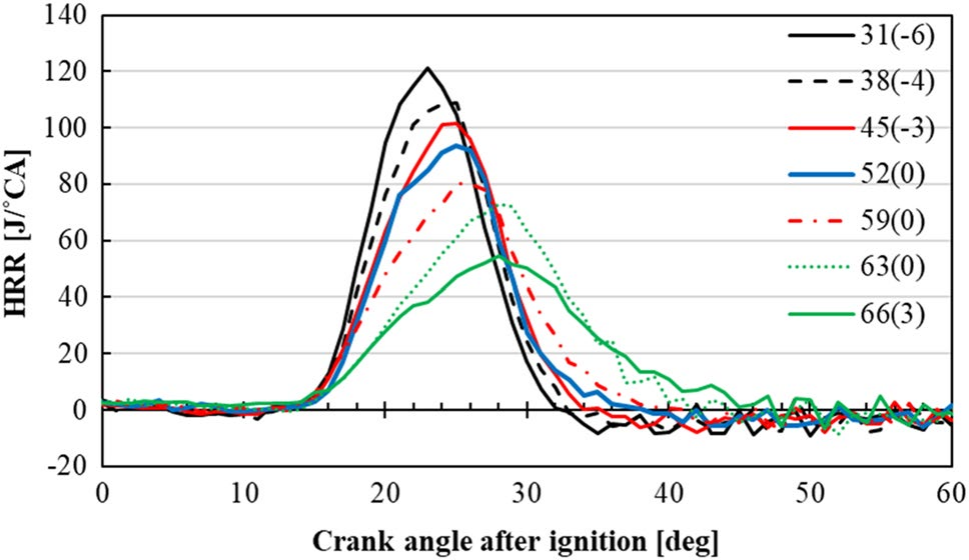
HRR for different NH_3_ content under the intake temperature of 348 K.

Figure 4 shows the change in heat release rate (HRR) after ignition at each MBT for the intake temperature of 348 K, calculated from Eq. (8). The ignition timing was normalized to 0°CA, as it was different on each condition due to different NH_3_ content in the fuel mixture. It should also be noted that, after peaking, the HRR asymptotically approaches zero, which indicates an accurate compensation for cooling loss (calculated from Eq. (4)) from the combustion chamber. From this figure, it became apparent that as the NH_3_ content got higher, HRR decreased. In addition, as can be seen from this figure, the width of each curve got wider as the NH_3_ content got higher. For NH_3_ content of 31%, the start and the finish of HRR was found to be 17°CA, while for NH_3_ content of 66% the width was increased to 30°CA. This result was thought to be related to the slow chemical kinetics of NH_3_, taking longer time for combusted flame to propagate and release its energy. The peak points of the curves were also retarded as a result of slower LBV of NH_3_. In addition, it was expected that HCCI combustion occurred in the main chamber, since the HRR curve is found to be similar to the previous study by Pochet et al.^7^.

**Figure 5:**
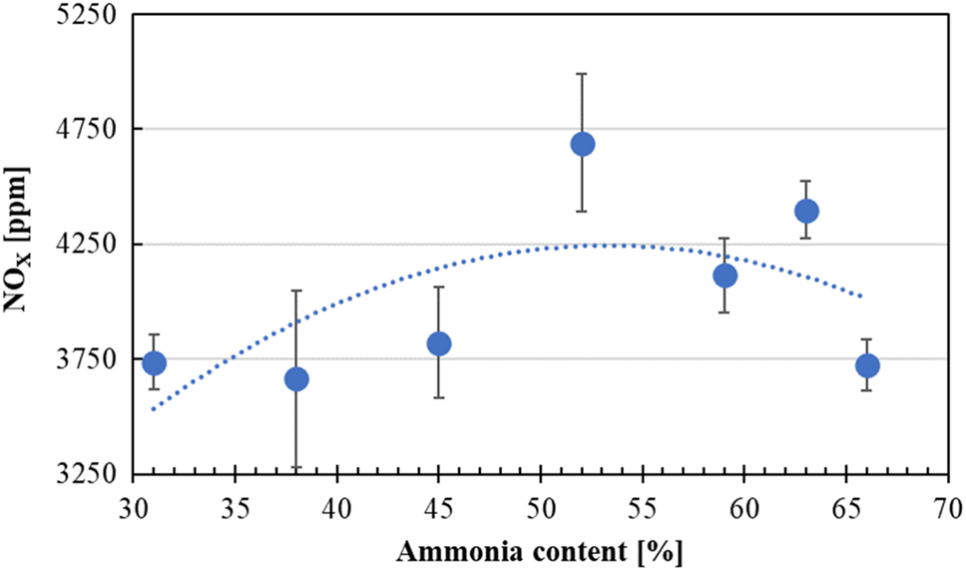
NO_X_ emissions at each MBT for various NH_3_ content of under the intake temperature of 348 K.

Figure 5 illustrates the NO_X_ emissions at each MBT for various NH_3_ content under the intake temperature of 348 K. From NH_3_ content of 31% to 52%, NO_X_ emissions showed an increasing trend. As the NH_3_ content further increased, NO_X_ emissions were lowered. This was related to the decreased combustion efficiency due to the delay in reaching high ignition temperatures for higher NH_3_ content cases. More details about this phenomenon are given in Fig. 9. With the increase of NH_3_ in the fuel mixture, due to the lower stoichiometric value of NH_3_, gasoline is left with an abundance of free oxygen molecules. This resulted in a leaner environment, creating difficult conditions for gasoline to burn to help NH_3_ to reach high ignition temperatures. Thus, in-cylinder gas temperature was decreased, which resulted in lowered NO_X_ emissions. However, when compared to previous studies^7–9^, where NH_3_ was used as an auxiliary or main fuel in engine experiments, current NO_X_ emission values were in-line with those results.

### Intake air temperature effect on co-combustion and engine performance

**Figure 6:**
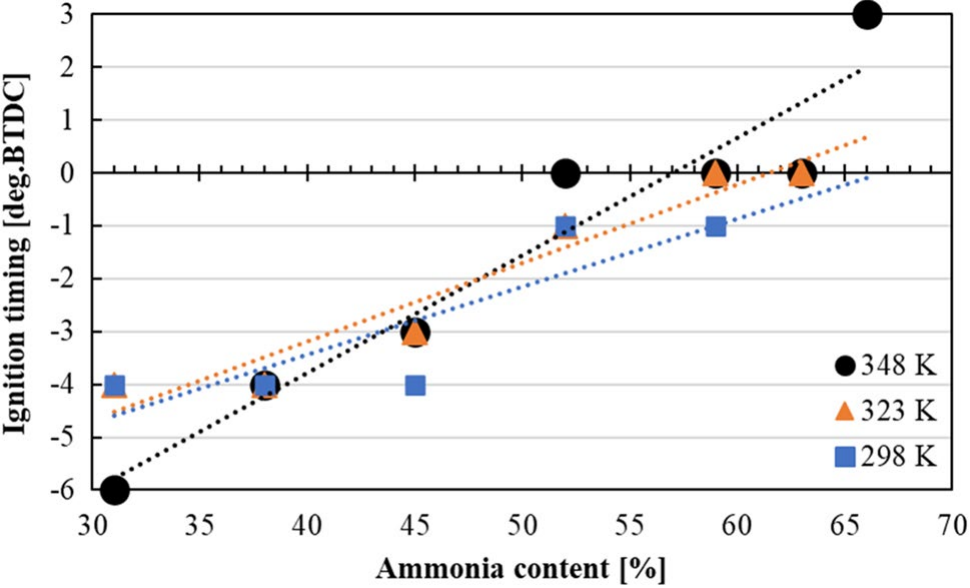
MBT of each NH_3_ content under intake temperatures of 298 K, 323 K and 348 K.

Figure 6 shows the influence of NH_3_ content on MBT advancement under different intake air temperatures. Since NH_3_ has slower LBV compared to gasoline (see Table 1), under the same intake air temperature, MBT was advanced (values going from − 6 to 3 deg BTDC) as the NH_3_ content was increased from 31 to 66%. In addition, the highest combusted NH_3_ content was increased with increasing intake air temperatures from 323 to 348 K. The reason for this result was thought to be the increase in the initial temperature of the air–fuel mixture promoted the low-temperature oxidation reaction which enables the ignition of both NH_3_ and gasoline fuels. Thus NH_3_/gasoline co-combustion range was extended.

**Figure 7:**
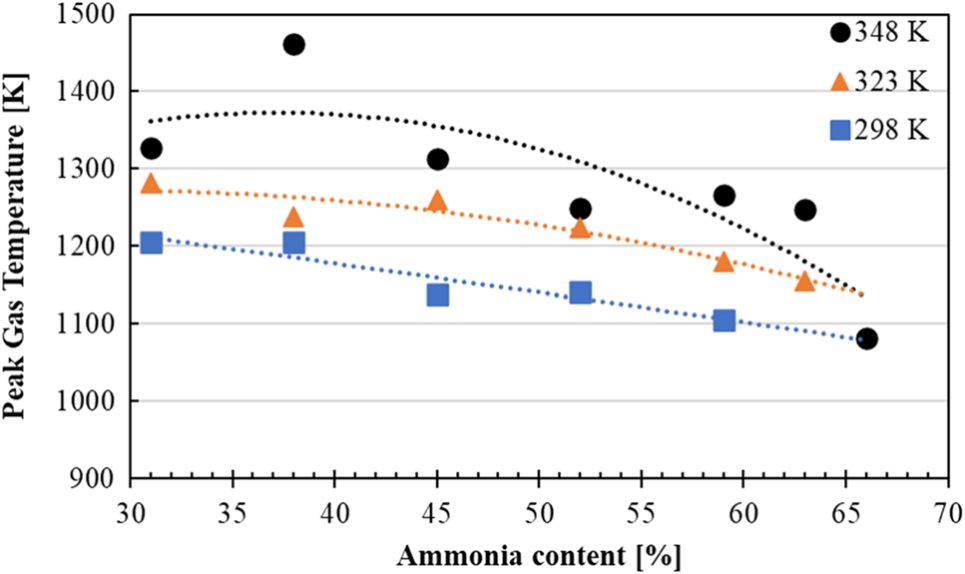
Peak gas temperature of each MBT condition at different NH_3_ content under the intake temperatures of 298 K, 323 K and 348 K.

Figure 7 shows the peak gas temperature change as the NH_3_ content was increased under different intake air temperatures, where air temperatures were calculated by Eq. (13). As expected, higher intake air temperature cases resulted in higher peak gas temperatures as the combustion completed. However, even though COV_IMEP_ values were still less than 5%, as the NH_3_ content was increased, at higher intake air temperatures the peak gas temperature was decreased at a faster pace. As the intake air temperature increased, low-temperature oxidation reaction was promoted, which also increased combustion gas temperature. However, as the NH_3_ content was further increased, combustion gas temperature was lowered due to both the delay in peak HRR and that in end of combustion. This is because of the slow combustion effect caused by the lean conditions as NH_3_ content increased.

**Figure 8:**
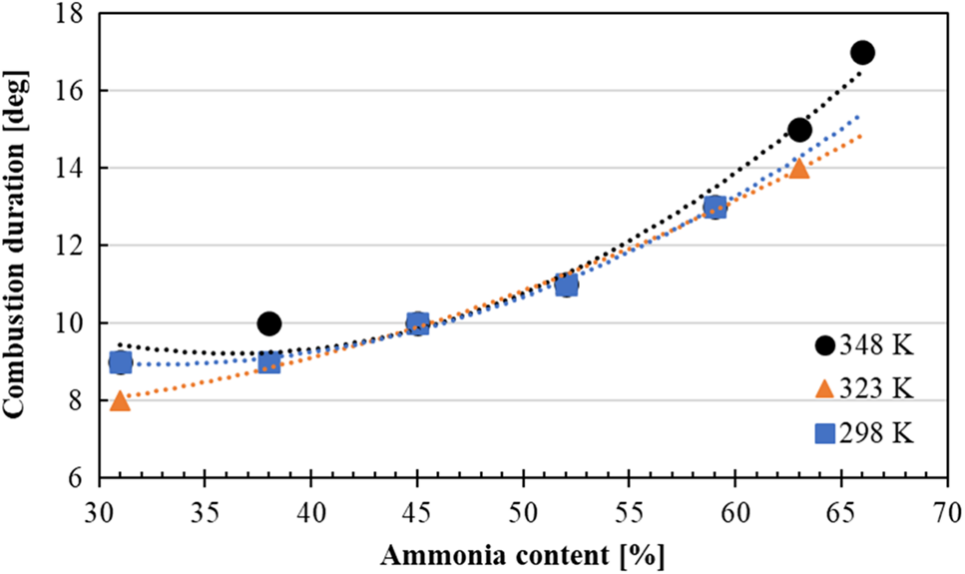
Combustion duration of each MBT condition at different NH_3_ content at the intake temperatures of 298 K, 323 K and 348 K.

Combustion duration is defined as the crank angle at which 10% (CA10) to 90% (CA90) of the total heat release energy is achieved in the modified engine. The combustion duration of each MBT under different intake air temperatures is shown in Fig. 8. At low NH3 content, higher intake temperatures showed shorter combustion duration, about 9°CA. However, as the NH_3_ content further increased, combustion duration started to increase, following a quadratic polynomial path. This was related to the slow kinetics of NH_3_, and leaner environment causing lower combustion efficiency for gasoline under higher NH_3_ content in the fuel mixture. However, it should be noted that with increased intake air temperatures NH_3_ content in the fuel mixture was increased to 66%, where the longest combustion duration was found to be around 17°CA. When the literature was reviewed, it was found that for a typical SI engine using gasoline, the combustion duration was calculated approximately to be around 26°CA^24^. These results show that the addition of a sub-chamber, which was equipped with spark and glow plugs, promoted the combustion mechanism even for NH_3_/gasoline fuel blends.

**Figure 9:**
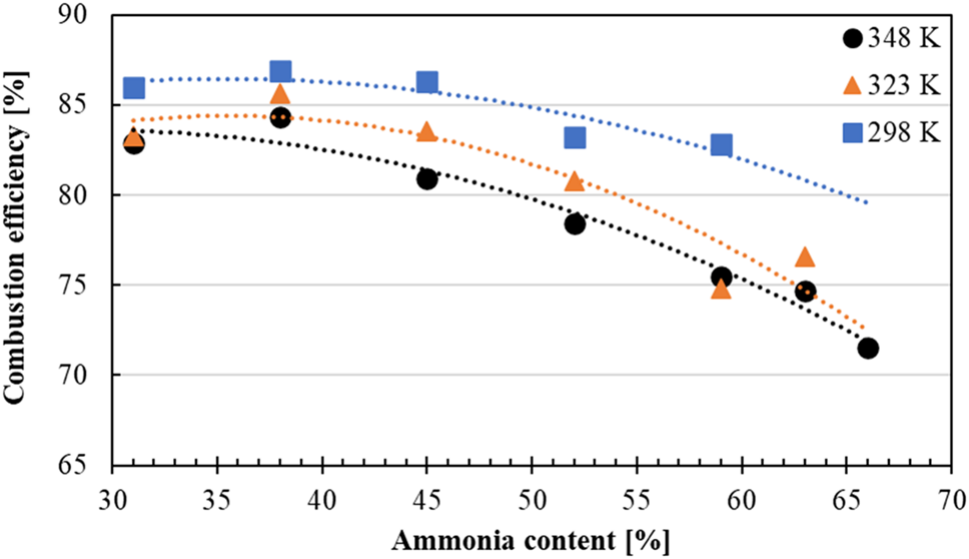
Combustion efficiency of each MBT at different NH_3_ content under the intake temperatures of 298 K, 323 K and 348 K.

Figure 9 illustrates the combustion efficiency, which was calculated by Eq. (9), under different intake air temperatures, with NH_3_ content increasing from 31 to 66%. Initially, at 31% of NH_3_ content, all intake air temperature conditions showed similar combustion efficiency values. However, as the NH_3_ content was increased, combustion efficiencies were lowered, especially for the higher intake air temperatures. This was thought to be related to the fact that NH_3_ has high ignition temperature, which was affected by decreased charging efficiencies at higher intake temperature cases. It should be noted that the excess air ratio was altered between 1.17 and 1.22, depending on the NH_3_ content in the fuel mixture. It was reported that oxygen-enriched conditions were more suitable for NH_3_ combustion, as LBV was increased^16^. However, this condition was not suitable for complete gasoline combustion. With these conditions, under the same intake air temperature, as the NH_3_ content was increased, the air–fuel mixture got leaner for the gasoline fuel. This was related to the lower stoichiometric air-to-fuel ratio mass needed for NH_3_ combustion. With increased NH_3_ in the fuel mixture, gasoline was left with an abundance of oxygen, resulting in a leaner environment. This excess air environment caused lower combustion efficiency for gasoline, when compared to the standard SI gasoline engine^25^. As a result of this phenomenon, without complete gasoline combustion, NH_3_ could not reach high ignition temperatures fast enough. Thus, further lowering the overall combustion efficiencies at higher NH_3_ content. Furthermore, the piston ring was found to be stuck with apparent corrosion on its surface when the experimental engine was inspected after the experiments. At the time of inspection, the engine timer showed 120 h. It is believed that this corrosion and the soot formation due to gasoline, caused the piston ring to get stuck and increased the ratio of blow-by gases during engine’s operation. With increased blow-by gases due to corrosion, some of the high temperature air/fuel mixture escaped to the crankcase, lowering the overall combustion efficiencies, under all intake air temperatures.

**Figure 10:**
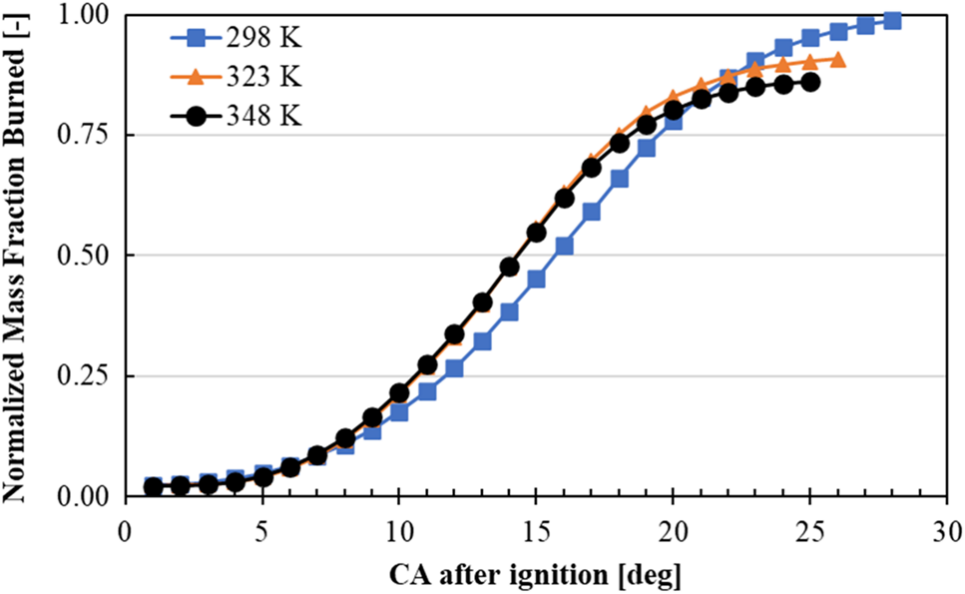
Intake air temperature effect on mass fraction burned and combustion duration @NH_3_ content of 59%.

Figure 10 illustrates the comparison of intake air temperature effect on normalized mass fraction burned results under 59% of NH_3_, which were calculated by Eq. (10). 59% was the highest NH_3_ content where stable comubustion was achieved under all intake air temperatures. As can be seen from the figure, when the intake air temperature was increased, combustion duration got narrower, which was an indication of high temperature enviroment promoting the NH_3_’s combustion speed. However, as the intake air temperature increased, mass burned fraction was lowered. This was thought to be an apparent result of lower charging efficiency causing incomplete combustion under higher intake air temperature cases.

**Figure 11:**
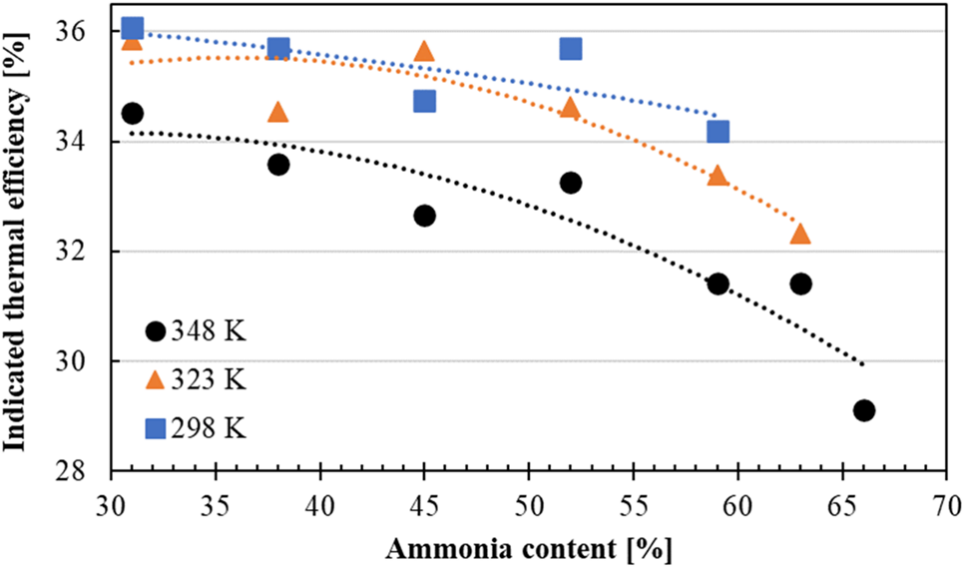
Indicated thermal efficiency of each MBT at different NH_3_ content under the intake temperatures of 298 K, 323 K and 348 K.

Figure 11 shows the indicated thermal efficiency, calculated by Eq. (12), results at different intake air temperatures as NH_3_ content was increased. As shown in Table 1, and in-line with previous studies^7–9,11^, in order to promote NH_3_ combustion, the ICE needs to have high compression ratio with higher ambient temperature inside the main-chamber for stable and more efficient combustion. However, elevated intake air temperatures resulted in loss in charging efficiency. Thus, it became apparent as the intake air temperature was increased, thermal efficiencies were decreased in a faster pattern for higher NH_3_ contents. In addition, similar to the combustion efficiency results, at the same NH_3_ content, higher intake air temperatures resulted in decreased charging efficiency, causing even lower indicated thermal efficiency results.

## Conclusion

In this study, a modified 17.7:1 CR spark-assisted CI engine with a sub-chamber equipped with glow and spark plugs was operated under various NH_3_/gasoline blend ratios. Glow plug and spark plug were used at the sub-chamber to increase the ambient temperature of the fuel mixture which would enhance the flame propagation speed of the fuel. Using a naturally aspirated system with an intake air heater mechanism, coolant temperature at 343 K, the modified experimental engine was able to operate with NH_3_ content of 66% under 1000 rpm. For all cases, COV_IMEP_ values were less than 5%, indicating a stable combustion process took place in the modified experimental engine. In addition to engine performance, corrosion was found on the piston ring. Several key findings from this study are listed as the following:

- It was confirmed that utilization of spark plug, and glow plug inside the sub-chamber of the modified engine enabled shorter combustion duration. This was thought to be an outcome due to the utilization of intake air heater, creating higher gas temperatures inside the combustion chamber promoting the combustion. The longest combustion duration was found to be around 17°CA, which was a drastic improvement for NH_3_ with slow kinetics.
- As the intake air temperature increased, this induced the NH_3_ content in the fuel mixture to reach up to 66%. As NH_3_ content was increased, MBT was observed to be advanced.
- Engine experiments started with gasoline fuel in lean state (excess air), and then NH_3_ was added gradually into the fuel-mixture. Gasoline was used as promoter to help NH_3_ to reach high ignition temperature. However, as the NH_3_ content was increased further, more oxygen became available for gasoline—creating a leaner environment due to lower stoichiometric A/F mass ratio of NH_3_. This caused gasoline combustion efficiency to deteriorate, which resulted in a delay in reaching high ignition temperatures for NH_3_ to ignite. Thus, a further decrease in combustion efficiency was observed at higher NH_3_ content.
- It was found that, at the same NH_3_ content, as the intake air temperature was increased, charging efficiency was lowered. This caused lower in-cylinder temperatures, which resulted in slower flame propagation, thus reducing the combustion, and indicated thermal efficiencies.
- During the inspection of the experimental engine after 120 h of operation, corrosion and soot formation were found on the piston ring. This caused the piston ring to be stuck, where blow-by gasses were increased. The increase in blow-by gases resulted in lower combustion and thermal efficiencies than usual operation of the engine.

Finally, we believe that experimental results from this study made it clear the need for increased boost pressure at the intake system, in order to improve the charging efficiency at higher intake air temperatures to further improve the performance and efficiency of the modified experimental engine. In addition, it became clear that corrosion phenomenon needs attention for long operation hours of the experimental engine. Based on these results, we will also investigate the relationship between corrosion in the combustion chamber and its effects on the combustion characteristics, which will be the main topic in our upcoming studies.

## Data Availability

The authors declare that all experimental data supporting this study are available from the corresponding author upon reasonable request.
